# Rectal malignant peripheral nerve sheath tumor with extremely high Ki-67 index (80%) and concomitant meningioma history: a case report

**DOI:** 10.3389/fonc.2026.1809228

**Published:** 2026-05-07

**Authors:** Liangchen Li, Hongxun Ruan, Zeming Zhao, Jiyuan Zhang, Shaoqing Fan, Guiying Wang

**Affiliations:** 1 Department of General Surgery, The Second Hospital of Hebei Medical University, Shijiazhuang, Hebei, China; 2 Department of General Surgery, The Fourth Hospital of Hebei Medical University, Shijiazhuang, Hebei, China

**Keywords:** abdominoperineal resection (APR), H3K27me3, Ki-67, rectal MPNST, SOX-10, sporadic

## Abstract

Malignant peripheral nerve sheath tumor (MPNST) is an extremely rare and highly aggressive sarcoma with a generally poor prognosis. We report a case of a 68-year-old female with a 10-year history of meningioma resection (sporadic MPNST, no NF1 family history or clinical manifestations) who presented with anal pain without typical rectal cancer symptoms. Pelvic MRI showed a lesion in the lower rectum, presenting a “target sign” on T2WI and high signal on DWI. Postoperative pathological and immunohistochemical findings confirmed MPNST (S-100 (+), SOX-10 (+), H3K27me3 (partial loss), Ki-67 (80%)). The patient underwent laparoscopic abdominoperineal resection (APR) following the total mesorectal excision (TME) principle, achieving R0 resection. The patient recovered uneventfully without perioperative complications, and long-term surveillance was recommended. This case highlights the diagnostic challenges of sporadic rectal MPNST with non-specific symptoms and extremely high proliferative activity, and provides evidence for the feasibility of laparoscopic APR in low rectal MPNST. It also underscores the need for individualized adjuvant therapy and long-term surveillance in high-risk cases.

## 1 Background

Malignant peripheral nerve sheath tumor (MPNST) is a rare sarcoma originating from Schwann cells or perineurial cells, accounting for 3% to 10% of all soft tissue sarcomas (1, 2). It is highly invasive, prone to local recurrence and distant metastasis, and carries a generally poor prognosis.

Approximately 50% of MPNST cases are associated with neurofibromatosis type 1 (NF1) (3). Mutations in the NF1 gene lead to loss of function of its encoded protein, neurofibromin. This loss results in constitutive activation of the RAS signaling pathway, thereby driving tumorigenesis. MPNST development often progresses through multiple stages—from plexiform neurofibroma to atypical neurofibroma, and finally to malignant transformation—involving sequential loss of tumor suppressor genes such as p16INK4A and TP53 (4).

MPNST occurring in the rectum is exceedingly rare, accounting for less than 1% of all MPNST cases, and most are associated with neurofibromatosis type 1 (NF1). Sporadic rectal MPNST (defined by no NF1 family history and absence of NF1 clinical manifestations) is even rarer, and cases complicated with a history of central nervous system tumors (e.g., meningioma) have not been reported in the literature to date (5). This unique phenotype further increases the difficulty of preoperative diagnosis and treatment decision-making, as its clinical manifestations (e.g., anal pain, blood on digital rectal examination) are non-specific and can mimic common rectal diseases, often leading to delayed diagnosis. Imaging studies [computed tomography (CT), magnetic resonance imaging (MRI)] aid in evaluating tumor extent, but definitive diagnosis relies on histopathology and immunohistochemical staining. Typical immunohistochemical features include focal positivity for neural markers such as S-100 and SOX-10, while loss of H3K27me3 has emerged as an important auxiliary diagnostic immunohistochemical marker (6).

Wide surgical resection (R0 resection) is the primary curative treatment for localized MPNST (7). For rectal MPNST, complete resection is particularly challenging due to the complex anatomy. The role of adjuvant radiotherapy and chemotherapy remains unclear, with limited efficacy. Recently, targeted agents against the RAS/MEK/ERK pathway and immune checkpoint inhibitors have shown potential in clinical studies, offering new directions for future treatment (8).

## 2 Case presentation

A 68-year-old female presented with a two-week history of anal pain. She reported no systemic symptoms, no subjective hematochezia, abdominal pain, distension, diarrhea, tenesmus, or rectal heaviness. She had a 10-year history of meningioma resection (no recurrence) and hypertension (controlled with nifedipine). She denied any family history of neurofibromatosis type 1 (NF1) or other genetic disorders/tumor syndromes. On physical examination, the patient was alert and cooperative with normal general condition. Her body temperature was 36.6°C, pulse 76 beats per minute, respiratory rate 18 breaths per minute, and blood pressure 132/84 mmHg. No yellowish skin stain, rash, petechiae, café−au−lait spots, or cutaneous neurofibromas were found. No superficial lymphadenopathy was detected in the whole body. The head, neck, chest, and heart examinations were unremarkable. The abdomen was flat and soft, with no tenderness, rebound tenderness, or palpable masses. Liver and spleen were not enlarged under the costal margin. Bowel sounds were normal. Digital rectal examination (knee−chest position) revealed a palpable mass at the 12 o’clock position, approximately 1 cm from the anal verge. The mass was tough and mobile, and fresh blood was noted on the examination glove. No abnormalities were found in the spine, extremities, and nervous system.

**Figure 1:**
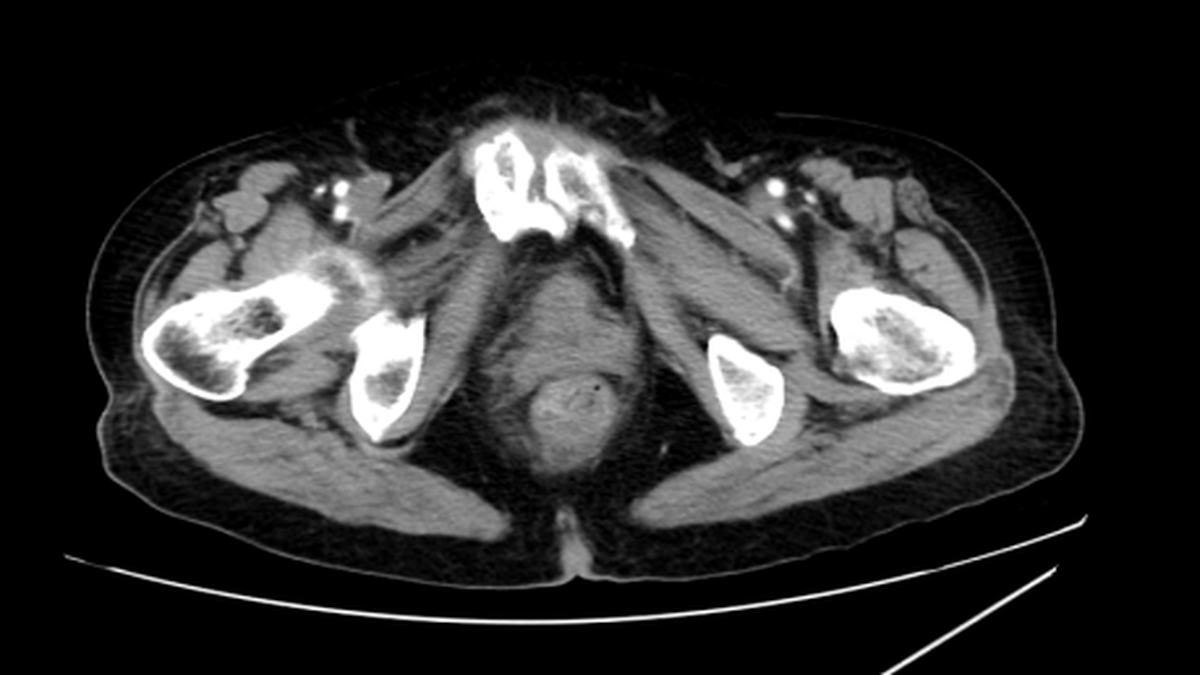
CT imaging characteristics. Distal rectal lesion.

**Figure 2:**
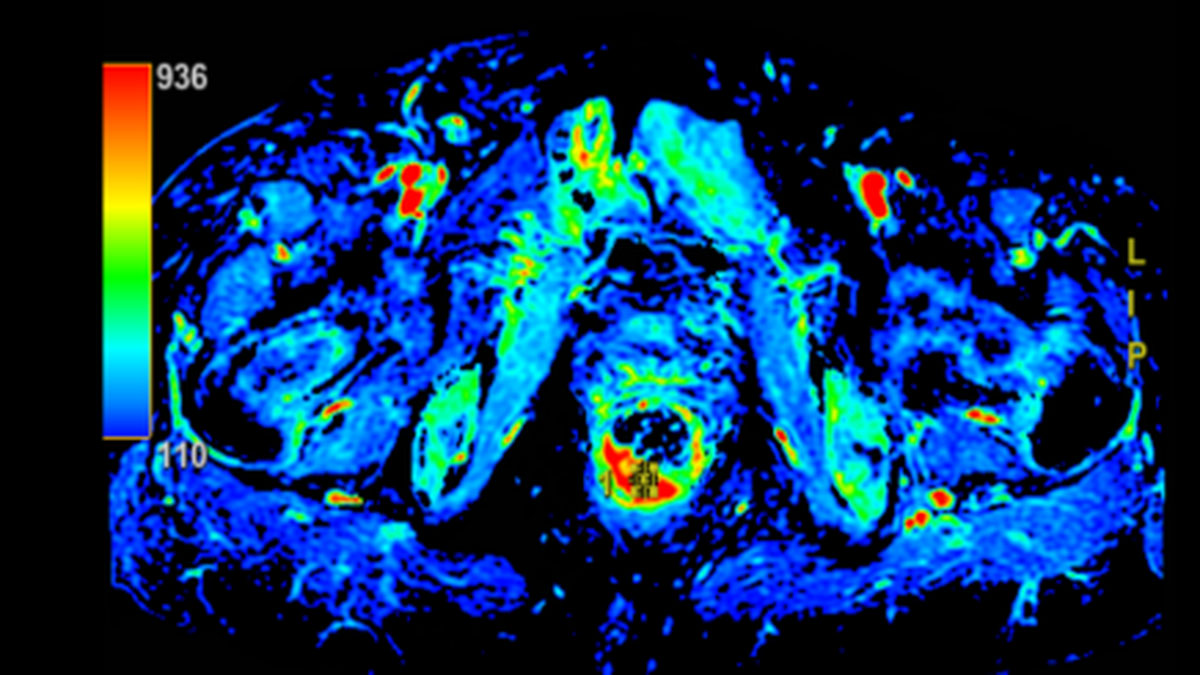
MRI imaging characteristics. Distal rectal lesion (target sign on T2WI, high signal on DWI).

**Figure 3:**
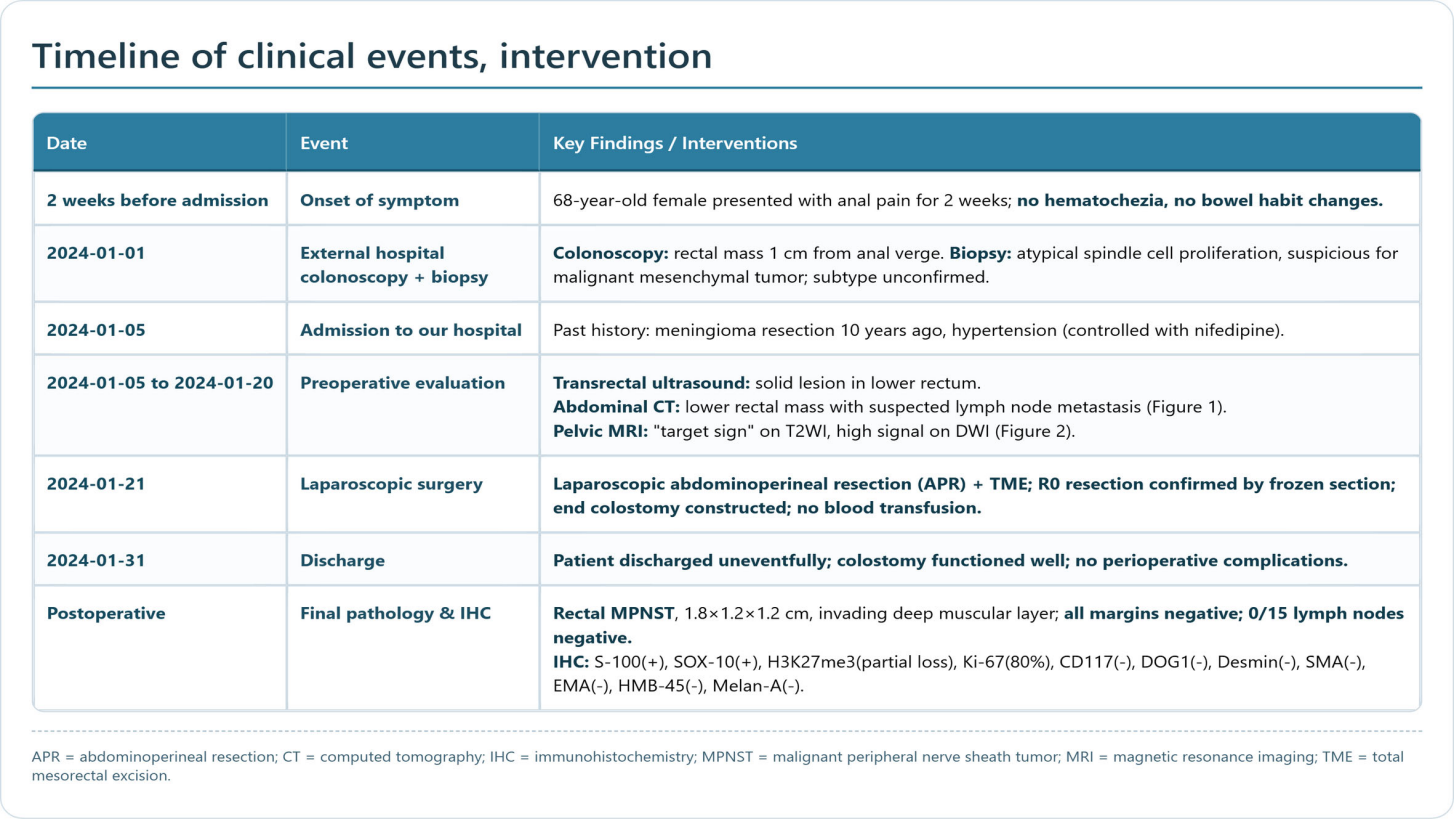
Timeline of clinical events and interventions for the patient with rectal MPNST.

Colonoscopy was performed at an external hospital, which identified a rectal tumor 1 cm from the anal verge. Pathological examination of the biopsy specimen showed atypical spindle cell proliferation, suggesting a malignant mesenchymal tumor and not excluding soft tissue-derived tumors, but failed to confirm the specific histological subtype. The patient was referred to our hospital for further management. Transrectal color Doppler ultrasound confirmed a solid lesion in the lower rectum. Abdominal CT showed a lesion in the lower rectum with suspected lymph node metastasis (**Figure 1**). Pelvic MRI demonstrated a “target sign” on T2WI and high signal on DWI (**Figure 2**). Postoperative pathological and immunohistochemical examination of the resected specimen in our hospital confirmed the diagnosis of malignant peripheral nerve sheath tumor. The diagnosis of sporadic MPNST was further supported by the absence of NF1 family history and NF1−related clinical manifestations, which excluded NF1−associated MPNST and fulfilled the diagnostic criteria for sporadic disease. The timeline of clinical events and interventions for this patient is summarized in the figure (**Figure 3**).

## 3 Treatment

After a comprehensive preoperative evaluation, exclusion of surgical contraindications, and multidisciplinary team (MDT) consultation, the patient underwent laparoscopic abdominoperineal resection (APR) under general anesthesia in January 2024, following the total mesorectal excision (TME) principle. Intraoperatively, a palpable mass 1 cm from the anal verge was identified, and high ligation of the inferior mesenteric vessels was performed. Intraoperative frozen section analysis confirmed negative circumferential resection margin (CRM) and distal margin. The sigmoid colon was transected, a proximal sigmoid colostomy was created in the left lower quadrant, and the anus was closed following transection of the anococcygeal ligament and levator ani muscles. No blood transfusion was required.

**Figure 4:**
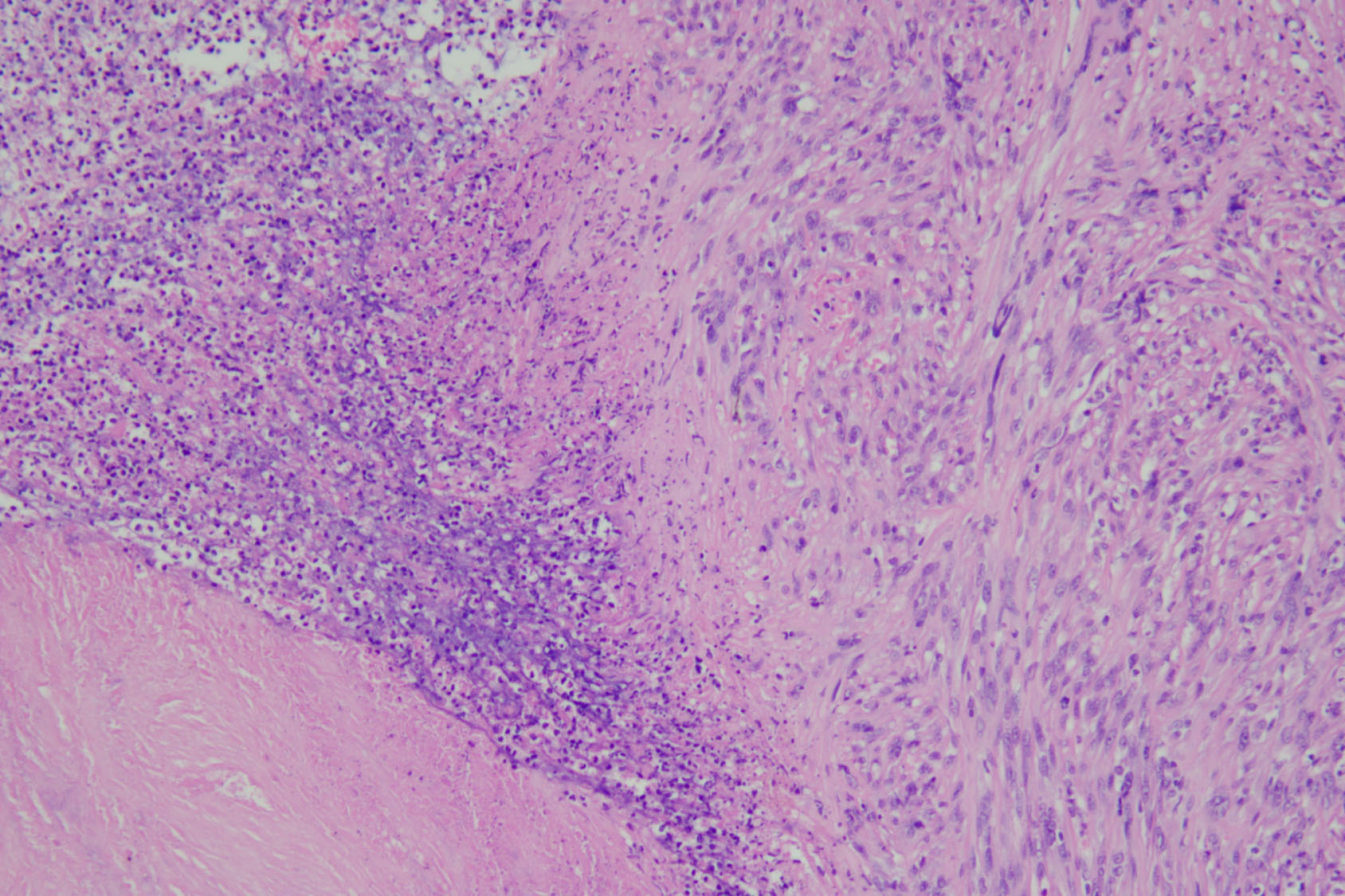
Histomorphological features of rectal MPNST. HE staining showed tumor cells invading the deep muscular layer of the rectal wall (×200).

The MDT discussed adjuvant therapy options: considering the limited efficacy of conventional radiotherapy and chemotherapy for MPNST (9), the patient’s history of meningioma resection (radiotherapy may increase central nervous system injury risk), and the complete R0 resection, it was decided to forgo adjuvant radiotherapy/chemotherapy and implement close follow-up. Pathological examination of the resected rectum and anal canal confirmed malignant peripheral nerve sheath tumor, consistent with its histomorphological and immunohistochemical features (**Figure 4**), measuring approximately 1.8 × 1.2 × 1.2 cm, invading the deep muscular layer of the rectal wall. No definite intravascular tumor thrombus or perineural invasion was identified. All resection margins (including the two residual margins of the specimen, circumferential resection margin, and dentate line) were free of tumor. No tumor metastasis was found in the highest lymph node group (0/3) or perirectal lymph nodes (0/12).

Immunohistochemistry results were as follows: H3K27me3 (partial loss), CD117 (-), CD34 (-), Desmin (-), DOG1 (-), Ki-67 (80%), S-100 (+), SMA (-), CKpan: scattered (+), Vimentin (+), EMA (-), INI1 (+), PR (-), SSTR2 (-), STAT6 (-), HMB-45 (-), Melan-A (-), SOX-10 (+). The final diagnosis was rectal MPNST. The patient recovered uneventfully after surgery. She was discharged on postoperative day 10 with a well-functioning colostomy (no ischemia, necrosis, or leakage) and well-healed abdominal and perineal incisions. No perioperative complications (e.g., bleeding, infection, intestinal obstruction) were observed. Given the high-risk features of this tumor (deep muscular invasion, Ki-67 index 80%), long-term close surveillance with pelvic MRI, abdominal CT, and tumor markers (CEA, CA19-9) every 3 months for the first 2 years is recommended.

## 4 Discussion

Rectal MPNST is an extremely rare, highly aggressive malignancy with a poor prognosis. While MPNST accounts for 3% to 10% of soft tissue sarcomas, primary rectal involvement is particularly uncommon. This report describes a 68-year-old female presenting with anal pain, with the tumor located in the lower rectum and definitively diagnosed as MPNST postoperatively via pathology and immunohistochemistry. This case illustrates the diagnostic, therapeutic, and prognostic challenges associated with this disease and offers valuable insights for clinical practice.

The clinical presentation of rectal MPNST is non-specific, often including anal pain, bleeding, or changes in bowel habits, which can lead to misdiagnosis as other common rectal malignancies (10). Given the overlapping histomorphological features of spindle cell tumors in the rectum, a comprehensive immunohistochemical panel is essential for definitive diagnosis, and the differential diagnosis should include the following entities:

### 4.1 Rectal adenocarcinoma

The most common rectal malignancy, typically shows diffuse and strong positivity for cytokeratin pan (CKpan). In our case, only scattered CKpan positivity was observed, which ruled out rectal adenocarcinoma.

### 4.2 Gastrointestinal stromal tumor

The most common rectal mesenchymal tumor, characterized by consistent positivity for CD117 and DOG1 (11). Both markers were negative in our case, excluding GIST.

### 4.3 Leiomyosarcoma

The most common primary rectal sarcoma, usually exhibits diffuse immunoreactivity for Desmin and smooth muscle actin (SMA) (12). Our case showed negative staining for both Desmin and SMA, which excluded leiomyosarcoma.

### 4.4 Synovial sarcoma

A rare spindle cell sarcoma that can occur in the rectum, often expresses epithelial membrane antigen (EMA). EMA was negative in our case, making the diagnosis of synovial sarcoma unlikely.

### 4.5 Undifferentiated pleomorphic sarcoma

A high-grade sarcoma that only expresses vimentin and lacks specific lineage markers. Our case showed strong positivity for neural markers S-100 and SOX-10, which excluded undifferentiated pleomorphic sarcoma.

### 4.6 Malignant melanoma

Amelanotic melanoma can present as a spindle cell tumor in the rectum, but it is characteristically positive for HMB-45 and Melan-A (13). Both markers were negative in our case, ruling out malignant melanoma.

Additionally, preoperative CT suggested lymph node metastasis, but postoperative pathology confirmed no metastasis in 15 lymph nodes (0/3 in the highest group, 0/12 perirectal), indicating a potential false-positive risk of imaging in rectal MPNST (14). This finding emphasizes the importance of pathological confirmation for accurate staging. In this case, imaging initially suggested a rectal mass, but definitive diagnosis required postoperative pathology and immunohistochemistry, which revealed characteristic features: S-100 (+), SOX-10 (+), partial loss of H3K27me3, and a markedly elevated Ki-67 index of 80%, although a ‘target sign’ on T2WI has been described in neurogenic tumors, it is not entirely specific for MPNST in the rectal location and may overlap with findings in rectal adenocarcinoma. H3K27me3 loss is an important epigenetic marker in MPNST; recent studies have indicated that loss of H3K27me3 is associated with poorer prognosis and may serve as a potential marker for targeted therapy. The exceptionally high Ki-67 index in this case (significantly higher than that in most soft tissue sarcomas) further supports high proliferative activity and correlates with an increased risk of adverse outcomes (15).This case highlights the significant diagnostic challenges of rectal MPNST. The non-specific clinical presentation and overlapping histomorphological features with other spindle cell tumors often lead to delayed diagnosis or misdiagnosis, especially in primary care settings. Prognostically, the deep muscular invasion and extremely high Ki-67 index (80%) in this case indicate a high risk of recurrence and metastasis, which necessitates intensive long-term surveillance. The partial loss of H3K27me3 further supports a more aggressive biological behavior.

Currently, complete surgical resection (R0) remains the only curative treatment for localized MPNST (16). This patient underwent laparoscopic APR, with postoperative pathology examination confirming negative margins and complete local excision. Achieving R0 resection for rectal MPNST is challenging due to the complex pelvic anatomy, and the success rate for complete excision is generally low. Despite advances in surgical techniques, the risk of local recurrence and distant metastasis remains high, particularly in cases with large tumor size, deep invasion, or high proliferation index.

Regarding adjuvant therapy, conventional radiotherapy and chemotherapy (e.g., doxorubicin combined with ifosfamide) have limited efficacy against MPNST (16). Recent research has focused on exploring novel systemic strategies. For example, combination therapy with CDK4/6 and MEK inhibitors has demonstrated synergistic antitumor effects in preclinical models and may promote immune cell infiltration, providing a rationale for combination with immunotherapy (e.g., PD-1/PD-L1 inhibitors) (16). In recent years, the application of targeted therapy and immunotherapy in MPNST has attracted increasing attention. For instance, PRMT5 inhibitors targeting MTAP deletion have demonstrated significant antitumor activity in MPNST models, providing a new direction for the treatment of MPNST (17). Additionally, LLS30, an inhibitor of the Galectin-1/Ras signaling pathway, has shown favorable antitumor effects in preclinical studies of MPNST, serving as a potential candidate drug for the targeted therapy of MPNST in the future (18). These developments offer new directions for treating patients with unresectable, recurrent, or metastatic MPNST.

Due to the lack of long-term follow-up data in this case, the long-term prognosis cannot be definitively evaluated. However, previous studies have shown that MPNST with Ki-67 >50% and deep tissue invasion has a high risk of local recurrence and distant metastasis, with a 5-year recurrence rate of 40%-60% (19). Therefore, long-term and intensive follow-up is crucial for such high-risk patients. Our report focuses on the diagnostic challenges and surgical feasibility of rectal MPNST, while the long-term outcomes require further collection and analysis of follow-up data in the future. Despite the lack of long-term follow-up, existing evidence on MPNST prognosis can still provide valuable references for clinical practice.

The overall prognosis for MPNST is poor, with a five-year survival rate of approximately 23% to 69% (20). Primary rectal MPNST may have an even worse prognosis due to its anatomical location and tendency to invade surrounding structures. Although this patient achieved R0 resection, the presence of deep muscular layer invasion and a high Ki-67 index necessitates long-term follow-up and imaging surveillance. The partial loss of H3K27me3 in this case suggests a higher-risk molecular subtype, warranting closer monitoring and consideration of more aggressive adjuvant strategies.

Notably, this is the first reported case of sporadic rectal MPNST with a history of meningioma resection in the English literature. This case reminds clinicians that during preoperative evaluation, in addition to routine NF1-related screening, the possibility of multiple primary tumors should also be considered. Although no established genetic link between meningioma and sporadic MPNST exists, this case highlights the importance of comprehensive surveillance in patients with a history of other primary tumors. This expands the clinical spectrum of rectal MPNST and provides new insights for preoperative assessment. As an extremely rare and highly aggressive tumor, the diagnosis of rectal MPNST relies on the combination of histomorphology and comprehensive immunohistochemical markers (e.g., S-100, SOX-10, H3K27me3) (21). Therapeutically, every effort should be made to achieve R0 resection, with adjuvant therapy considered based on individual risk stratification. In the future, with deeper insights into its molecular mechanisms, targeted therapies against the RAS/MEK/ERK pathway, immune microenvironment, and epigenetic regulation, as well as immunotherapies, are expected to bring new hope to these patients. Meanwhile, establishing a multidisciplinary management model and promoting molecular subtype-guided individualized treatment strategies will be crucial for improving patient outcomes.

This case report has several clinical strengths. First, it reports a very rare case of primary sporadic malignant peripheral nerve sheath tumor (MPNST) of the rectum, which is rarely reported in clinical practice. Second, the complete clinical data including external examination, preoperative evaluation, surgical procedure, postoperative recovery and detailed pathological and immunohistochemical results are provided, which can provide reference for clinical diagnosis and treatment of similar rare tumors. Third, this case confirms that laparoscopic abdominoperineal resection can achieve safe R0 resection for low rectal MPNST, which has certain clinical guiding significance.

However, this study also has some limitations. First, this is a single-case report from a single center, and the relevant conclusions cannot be extended to all patients with rectal MPNST. Second, no molecular genetic detection was performed, so the potential molecular mechanism of tumorigenesis cannot be further analyzed. Third, long-term follow-up data are not available, so it is impossible to evaluate the long-term prognosis such as recurrence and metastasis.

## 5 Conclusion

Rectal MPNST is an extremely rare and highly aggressive tumor, with diagnosis relying on the combination of typical imaging features (e.g., MRI target sign), comprehensive immunohistochemical profiling (S-100, SOX-10, H3K27me3). Laparoscopic APR following the TME principle is a feasible option for low rectal MPNST to achieve R0 resection. For sporadic cases with extremely high Ki-67 index and concurrent history of other tumors, close long-term follow-up (every 3 months for the first 2 years) is essential, and adjuvant therapy should be individualized based on MDT consultation. This case enriches the clinical data of rare rectal MPNST phenotypes and provides valuable insights for diagnosis and treatment optimization.

## Data Availability

The original contributions presented in the study are included in the article/supplementary material. Further inquiries can be directed to the corresponding author.
